# Surface Modification by the DBD Plasma to Improve the Flame-Retardant Treatment for Dyed Polyester Fabric

**DOI:** 10.3390/polym13173011

**Published:** 2021-09-06

**Authors:** Ha-Thanh Ngo, Khanh Vu Thi Hong, The-Bach Nguyen

**Affiliations:** School of Textile Leather and Fashion (STLF), Hanoi University of Science and Technology (HUST), No. 1, Dai Co Viet, Hai Ba Trung, Hanoi 11517, Vietnam; thanh.ngoha@hust.edu.vn (H.-T.N.); nguyenbach28095@gmail.com (T.-B.N.)

**Keywords:** DBD plasma, flame-retardant, dyed PET fabric, pad-dry-cure method, LOI, tensile strength, CETAFLAM PDP 30

## Abstract

In the first part of the study, dyed polyester fabric was treated with a dielectric barrier discharge (DBD) plasma at 1 W/cm^2^ for 15, 30, 60 and 90 s. The wicking height, tensile strength and color of the control and plasma treated fabrics were measured. Results show that the fabric capillary increases with plasma treatment time up to 90 s. However, plasma treatment time longer than 60 s caused an obvious color change and decrease in tensile strength of fabric. Plasma contact time should be such that plasma can improve the hydrophilicity of the fabric and adversely affect the properties of the fabric as little as possible. Thus, the suitable plasma contact time should be less than 60 s. Based on these results, in the second part of the study, three different time levels (15, 20 and 30 s) were selected for plasma pretreatment of this fabric. The plasma-treated fabric was then padded with the flame retardant (FR) (CETAFLAM PDP 30), dried and finally cured at 190 °C for 120 s. The limited oxygen index (LOI) of FR fabrics and the vertical fire characteristics of FR fabric after being washed 5 times also were measured. Comparison of these results with those of FR fabrics without plasma pretreatment shows that plasma pretreatment improves the fabric’s flame retardancy and FR durability. Moreover, it also reduces the heat shrinkage of PET fabric due to high temperature curing. The scanning electron microscopy (SEM) images of the fabric after plasma treatment and FR treatment and the energy-dispersive spectroscopy (EDS) spectrum of the fabric are consistent with the above results.

## 1. Introduction

Poly(ethylene terephthalate) (PET) fiber has many desirable properties, such as high tensile strength, dimensional stability and resistance to many chemicals, that give it a wide range of applications. However, PET does not exhibit reactive functionalities, such as NH_2_, COOH, and/or OH groups wanted for different interactions, which gives the molecule of PET limited reactivity and innately hydrophobic [1]. Moreover, being a thermoplastic polymer, polyester melts easily, decomposes readily and burns when heated due to its low flame retardancy [2]. The traditional approaches in imparting FR properties to the synthetic textiles, especially at the fiber stage, involve the addition of halogen and/or phosphorus-containing comonomers to the polymer structure during copolymerization [3]. Considering the adverse impacts of fiber stage finishing on the physical properties of modified textiles, a form of topical or surface finishing or post-treatments onto the fabric surfaces is commonly chosen for conferring the flame retardancy of synthetic textiles. The traditional method of fabric surface treatment is a pad-dry-cure technique, in the form of finishes and coatings [2,3]. However, the hydrophobic nature of polyester results in a relatively low chemical absorbance during the finishing treatment [4]. Surface treatment of polyester fabrics to increase the hydrophilic properties of the fabric is a solution that has been implemented in many studies. The traditional surface treatments commonly applied to PET fabrics are often chemical treatments [5]. The most common is the treatment of PET fabrics with strong alkalis in high concentrations and at high temperatures [6,7]. Alkaline treatment for polyester fabric leads to cleavage of the ester bonds on the surface of the fiber. The result is the formation of terminal hydroxyl and carboxylase groups on the fiber surface. Hydrolysis is believed to be increase the number of polar functional groups in the fiber surface [5]. However, the work in [6] has also shown that treating polyester fabric with sodium hydroxide solution reduces the tensile strength and weight of the fabric, allowing improvement in the fabric’s hand feel. These effects increase with increasing sodium hydroxide concentration, time, and temperature of treatment [5]. Another chemical treatment used on PET fibers is amine treatment [5,8]. These chemicals can introduce the amine functional groups and increase wettability and anionic dyeability of the PET fabric as well as comfort of the fabric by sacrificing about 5–20% of tensile strength and 1% to 2% of weight, while alkaline hydrolysis can reduce tensile strength by 5–10% and weight by 10–15% [8]. Thus, although chemical treatment of PET fabric has been effective in terms of the hygroscopicity and aesthetics of the fabric, it is often accompanied by a decrease in the mechanical strength and weight of the fabric. Furthermore, the environmental consequences should also be considered when using these methods.

From the above reasons, in recent years, non-thermal plasma techniques for surface modification of PET fabrics have been investigated by many researchers [9,10,11,12,13,14]. Plasma is partially ionized gas, composed of highly excited atomic, molecular, ionic and radical species with free electrons and photons. It is the interactions of these excited species with solid surfaces placed in the plasma environment that leads to the chemical and physical modifications of the material surface [12,15,16]. The advantage of plasma is that it allows modification of the surface of the material both physically and chemically without changing the bulk properties of the material [9,10,12,15,16]. In the textile sectors, the dielectric barrier discharge (DBD) technology is one of the most efficient non-thermal plasma sources in the atmosphere due to its scalability to large systems [16,17]. DBD is a class of plasma source that has an insulating (dielectric) cover over one or both of the electrodes and operates with high voltage power [16,17,18,19]. The plasma in DBD is cold because of the presence of the dielectric layer, which limits the heating current in the circuit while the displacement current has not any heating effects [16,19]. Due to these advantages, the DBD plasma is widely applied to surface treatment of textiles, especially polyester fabrics. The works of [9,11,12,13] have shown that DBD plasma (oxygen, or in the air) leads in appearance of new polar functional groups such as COO-, COOH, OH on a polyester surface. Physically, these works also show that certain lamellar structures have appeared on the fiber surface. These surface modifications have allowed improvement of wettability of polyester fabrics.

Due to the aforementioned effects, researchers have applied plasma to activate polyester fabric before the FR treatment of fabric. Raslan et al. [19] (2011) prepared PET textiles with FR properties using aluminum oxide (Al_2_O_3_) via DBD air plasma treatment. It is explored that the air plasma treatment with Al_2_O_3_ improves the flame retardancy of the fabric. Moreover, the TGA reveals that this form of treatment can improve the thermal stability of fabric. Carosio et al. [20] also used cold oxygen plasma at a pressure of 50 Pa at different processing conditions of time (15, 60 and 180 s) for surface activation of PET fabric before adsorbing nanoparticles onto the PET fabric surfaces, in order to improve the thermal stability and FR properties. The combustion behavior explores that the plasma activated fabrics have a remarkable improvement in time to ignition (up to 104%) and have a slight reduction in heat release rate (ca. 10%) as compared to the neat PET. In work [4], to reduce the quantity of chemicals required to produce FR polyester fabrics, a low-frequency oxygen plasma treatment was performed for 10 min before padding the polyester fabrics with alkyl-phosphonate-structured FR agents. The results have shown that the hydrophilic properties of the polyester fabrics improved after oxygen plasma treatment. Moreover, the consumed chemical amount was half of the maximum concentration without the LOI changing significantly. In work [21], an organophosphonate (OP), as the FR agent, was applied to the untreated and oxygen plasma-treated polyester fabric samples using the pad-dry-cure method. The results reveal that oxygen plasma treatment prior to finishing significantly increased the wettability of the polyester fibers, which directly resulted in increased concentration of the absorbed finishing agents. This allows the plasma pretreated sample to have better FR properties than those of the control sample. Thus, works [4,19,20,21] have shown that the plasma pretreatment for polyester fabric improves the efficiency of the subsequent flame retardant treatment despite using different types of plasma and flame retardants. However, plasma pretreatment for PET fabric can also have undesirable effects. Work [12] has shown that the DBD plasma treatment reduced the burst strength of the fabric by 14–30%, depending on the treatment conditions. Besides that, for the atmospheric plasma, the plasma treatment times were too long (15 min for [9], 30 min for [11], and 5 min for [12]). This would make it difficult to develop research results on an industrial scale. The aforementioned studies (except study [12]) used undyed PET fabrics, as their purpose was to activate the PET fabric prior to dyeing or printing [9,11,13]. The works that applied the plasma to activate polyester fabrics before the FR treatment [4,19,20,21] also used undyed fabrics. Meanwhile, the functional finishing treatment for the fabric is usually performed after the dyeing step. Therefore, in order for the PET fabric to be activated before the finishing step, the plasma treatment needs to be performed on the dyed fabric. These remaining issues will be elucidated in this study. In the first part of this study, the short-time DBD plasma treatments (from 15 to 90 s) are performed on the dyed polyester fabric, and the changes in the surface properties of the fabric after plasma treatment are analyzed. Based on these results, plasma treatment time for the second study (plasma activation effect on flame retardant treatment for dyed woven fabrics) was selected. The first goal of this study is to find the effective plasma contact time for the dyed woven polyester fabrics. It should be such that plasma can improve the hydrophilicity of the fabric and adversely affect the properties of the fabric as little as possible. The second goal is to clarify the effect of DBD plasma pre-activation (with selected exposure time) on the subsequent flame-retardant treatments. The first new feature of this study is the effects of DBD plasma in the short term on the surface modification of the dyed woven polyester fabric. The second novelty is the impact of this DBD plasma pretreatment on the effectiveness of the subsequent flame-retardant treatments for dyed woven polyester fabrics.

## 2. Materials and Methods

### 2.1. Materials

**Figure 1:**
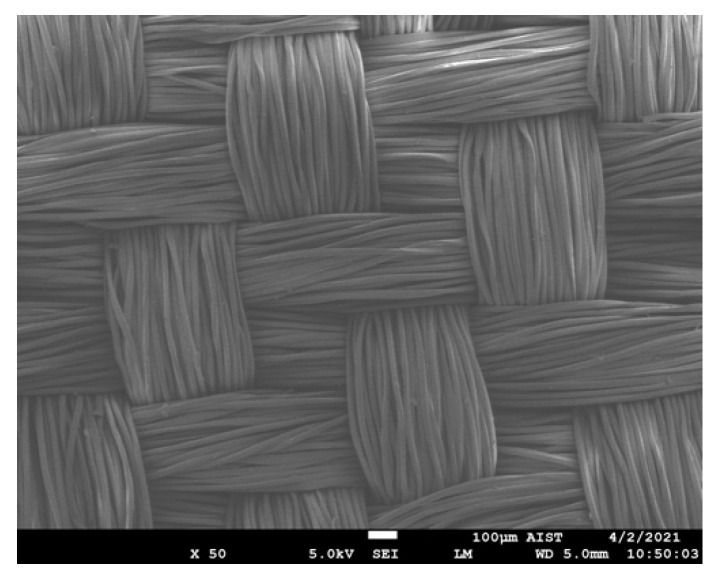
Scanning electron microscope (SEM) image (50× magnification) of 100% dyed polyester woven fabric.

Fabric: The 100% dyed polyester woven fabric was supplied by NASILKMEX (Nam Dinh, Vietnam). It is 3/1 twill weave fabric composed of filament yarns with the construction of 37 × 37 (Tex)/300 × 240 (per 10 cm), weighing 203 g/m^2^. The color of the fabric is light gray (Figure 1). The dyeing was at 130 °C for 40 min.

Chemicals: CETAFLAM PDP 30 was supplied and provided by Avocet Dye & Chemical Co. Ltd. (Brighouse, United Kingdom). Avocet manufactures a range of high performance, textile flame retardant chemicals marketed under the name CETAFLAM^®^. CETAFLAM PDP 30 is an organo-phosphonate ester flame retardant. It is a non-halogen flame retardant used in padding method for polyester fabric. Ammonia was used to adjust the pH of solution.

### 2.2. Methods

#### 2.2.1. Flame-Retardant Treatment for the DBD Plasma-Treated Dyed Polyester Fabric

##### 2.2.1.1. Treatment Procedure

**Figure 2:**
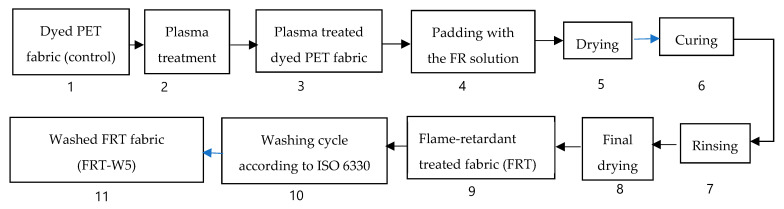
Treatment procedure for the dyed polyester fabric.

The dyed polyester fabric was treated following the procedure described in Figure 2.

In this study, the dyed PET fabric was treated following 3 different procedures:

Process 1 (From 1 to 3)—The surface modification for the dyed PET fabric by DBD plasma. The dyed PET fabric is treated with the DBD plasma.

Process 2 (From 1 to 9)—The FR treatment for the plasma-treated dyed PET fabric (PET fabric). The products of this process are flame retardant-treated fabric (FRT fabric).

Process 3 (From 1 to 11)—After step 9, FRT fabrics are then washed 5 times according to ISO 6330 to assess the washing fastness of their FR properties.

##### 2.2.1.2. DBD Plasma Treatment for Dyed PET Fabric

**Figure 3:**
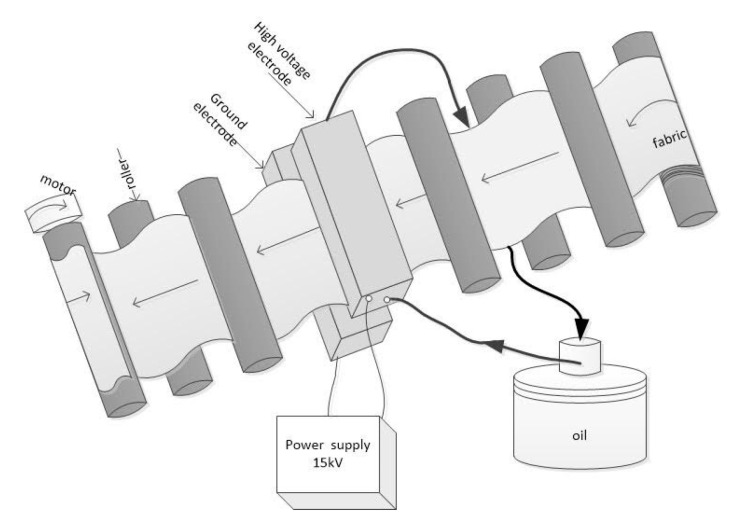
Schematic diagram of roll-to-roll DBD plasma system.

**Figure 4:**
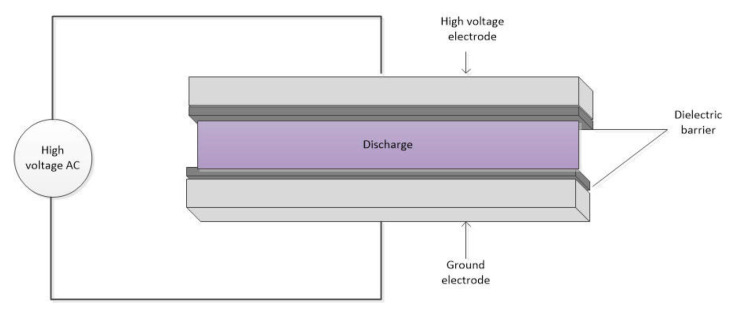
Schematic diagram of the atmospheric pressure DBD cell.

**Figure 5:**
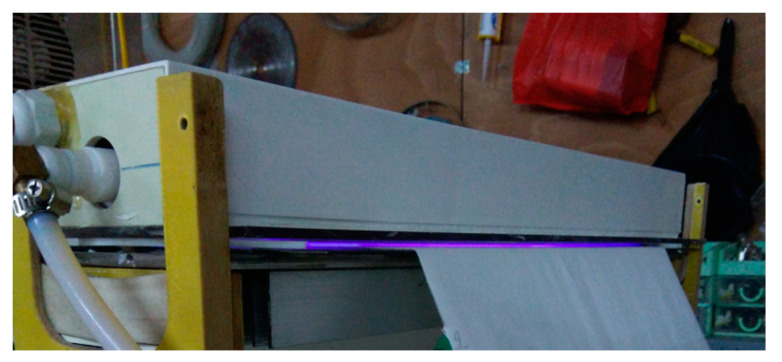
Photograph of air dielectric barrier discharge.

Laboratory atmospheric pressure DBD plasma equipment developed by the School of Engineering Physics (SEP) of HUST was used in this study. The schematic diagram of roll-to-roll DBD plasma system is shown in Figure 3. According to Figure 3, fabrics with a width of less than 50 cm can move continuously between the top and bottom electrodes. The tension rollers are placed before and after the electrodes to keep the fabric in even tension. The movement speed of the fabric can be controlled by a motor. Schematic diagram of the atmospheric pressure DBD cell is shown in Figure 4. According to Figure 4, DBD plasma is generated between two parallel electrodes, which are covered by a polycarbonate sheet as a dielectric layer. The thickness of the polycarbonate sheet for the high voltage electrode is 5 mm and that of the ground electrode is 3 mm. The length and width of the electrodes are 8 cm and 50 cm, respectively. The electrodes are connected to an AC source power supply whose frequency is 50 Hz and output voltage is 15 kV. The high voltage electrode is cooled by a circulated oil system, which is operated by a pump. The air DBD plasma (Figure 5) is generated in air as working gas under atmospheric pressure. In this study, the width of the dyed PET fabric was of 35 cm, the distance between the polycarbonate sheets (discharge gap) was 3 mm, the plasma treatment power was 400 W (1 W/cm^2^), an air atmosphere was employed, the plasma exposure times were: 15, 30, 60, and 90 s. Following those different plasma exposure times, the plasma-treated samples were marked as: P15, P30, P60 and P90, while the untreated sample (plasma exposure time = 0) was marked P0.

##### 2.2.1.3. Flame-Retardant Treatment for the Plasma-Treated Dyed Polyester Fabric

Polyester has no active groups that can form covalent bonds with FRs. Therefore, in order to have a durable FR fabric by pad-dry-cure method, generally, it needs to be cured at a high temperature for a long time [2]. Our survey research has shown that with the pad-dry-cure method, it is necessary to extend the curing time to 240 s at temperatures higher than 180 °C to achieve the required fire resistance for the FRT dyed polyester fabric after 5 washing cycles. However, 240 s is too long for an industrial process. For the above reasons, in this study, the dyed polyester fabric was pre-activated with DBD plasma before FR treatment. It is hoped that by plasma pre-activation, FR polyester fabric will have the required FR durability with the curing condition at 190 °C for 120 s (a suitable condition for industrial deployment). The plasma processing conditions were selected based on the research results of Section 2.2.1.2. It should make the dyed polyester fabric well activated and its negative influence on the physical and mechanical properties of the dyed polyester fabric should be as little as possible.

Based on the research results of Section 2.2.1.2, a set of experiments with different plasma exposure times (15, 20, and 30 s) were conducted for the dyed PET fabric and FR finishing with the selected curing condition (190 °C, 120 s). Besides this, another series that was not pretreated by plasma was also conducted to evaluate the effects of plasma pretreatment on FR treatment of fabric. The details of these experiments are present in Table 1.

Finishing solutions included: 750 mL CETAFLAM PDP 30, 220 mL H_2_O, and 30 mL ammonia 20% was used to adjust the pH of solution in the range of 6–6.5.

The plasma-treated fabric samples of 35 cm × 35 cm were impregnated with the finishing solution, then padded with the wet pick-up of approximately 75–80% by padder SDL D394A (SDL Atlas China). The padded samples were dried at 120 °C for 3 min. Afterward, these samples were cured at the conditions according to the options presented in Table 1. Stenter SDL D398 (SDL Atlas China) was used for the drying and curing steps. Next, the samples were rinsed under running cold water for 5 min to remove all the residual FRs on the fabric surface and neutralize the treated fabric. Then, the fabric was dried in the stenter at 120 °C for 3 min. The samples after FR treatment were named: P0-FRT1, P0-FRT2 P15-FRT, P20-FRT, and P30-FRT (Table 1).

##### 2.2.1.4. Washing for the FRT Samples

To determine the flame-retardant durability of the FRT fabric, the FRT samples were washed with 5 wash–drying cycles in accordance with ISO-6330 standard clause 6A. The Electrolux EW 1290W front load washing machine was used (Electrolux Vietnam). The samples after washing were named: P0-FRT1-W5, P0-FRT2-W5, P15-FRT-W5, P20-FRT-W5 and P30-FRT-W5.

#### 2.2.2. Characterization of the Control and Treated Dyed Polyester Fabric

The control, plasma-treated, FRT samples, and the FRT samples after washing were stored inside the polyethylene bags at the standard laboratory conditions for at least 24 h before any further analysis. All of the following experiments were conducted at the Testing center for textiles and leather materials of STLF (Hanoi, Vietnam).

##### 2.2.2.1. Characterization of the Control and Plasma-Treated Dyed Polyester Fabric

1. Tensile Properties of Fabrics

The ISO 13934–1:2013 standard method was used to determine the maximum force (F_max_) of the control and DBD plasma-treated samples. The tensile-testing machine Tenso Lab 2512A, Mesdan Lab (Brescia–Italy) was used for these tests. Using the Annex B of the ISO 13934–1: 2013 standard to prepare the samples: 50 ± 0.5 mm and 300 ± 0.5 mm, respectively, were the width and the length. The experiments were conducted with a gauge length of the tensile-testing machine of 200 ± 1 mm. The rate of extension of the tensile-testing machine was set to 100 mm/min. The mechanical strength change (MSC) of the fabric due to the DBD plasma treatment was calculated according to Equation (1).

(1) $$\mathrm{MSC}\;\left(\%\right)=\frac{F_{\max}\;\mathrm{of}\;\mathrm{untreated}\;\left(N\right)-{\mathrm{Calculated}\;F}_{\max}\;\mathrm{of}\;\mathrm{treated}\;\left(N\right)}{F_{\max}\;\mathrm{of}\;\mathrm{control}\;\left(N\right)}\times 100$$

The calculated F_max_ of the treated samples in Equation (1) is calculated based on the number of yarns of the control sample and the number of yarns of treated sample, Equation (2).

(2) $${\mathrm{Calculated}\;F}_{\max}\;\mathrm{of}\;\mathrm{treated}{\left(N\right)}^{}={\;\mathrm{Tested}\;F}_{\max}\;\mathrm{of}\;\mathrm{treated}\left(N\right)\frac{\mathrm{Number}\;\mathrm{of}\;\mathrm{yarns}\;\mathrm{of}\;\mathrm{control}}{\mathrm{Number}\;\mathrm{of}\;\mathrm{yarns}\;\mathrm{of}\;\mathrm{treated}\;}$$

2. Capillary Property of Fabrics

The capillary test was measured in accordance with TCVN 5073-1990 standard method. The experiments were conducted on the conditioned samples (65 ± 4%; 20% ± 2% at least for 24 h) with dimensions of 250 mm × 50 mm at the standard environment (65 ± 4%; 20 ± 2%). A total of 3 trips in the warp and 3 in the weft direction of the fabric were conducted. The conditioned samples were hung vertically with the end side immersed in potassium dichromate (1 g/L). The height of the fabric immersed in potassium dichromate was 10 mm. After each 5 min, up to 30 min, the wicking height at the center of the strip above the solution level was measured. The final result was the average value of 3 specimens.

3. Color Measurement

The colors of the control and plasma-treated samples were measured by Ci4200 Spectro-Photometer, produced by X-Rite Pantone-Michigan-USA (STLF, Hanoi, Vietnam with D65 illuminant and 10° standard observer. The measurement was repeated 3 times for each sample at 3 different positions. If the color difference between them is less than 0.35, the mean of the 3 measurements will be accepted as the color of the sample. The effect of plasma treatment on the fabric color is represented by the color difference (DE) between the control and plasma-treated sample. It can be expressed as DE_CIELab_ or DE_CMC_ values [22]. In this study, DE_CIELab_ and DE_CMC_ were calculated based on the color parameters of the samples by the software of Ci4200 spectrophotometer. The color strength (K/S values) of the samples from 400 to 700 nm were also established according to the Kubelka–Munk Equation [23] from the measured reflectance values (R). K/S value plots from 400 to 700 nm of the samples are also used to evaluate the effect of plasma treatment on the color of dyed PET fabrics. The color intensities of the samples were determined at the wavelength corresponding to the maximum absorbance (K/Smax) [23,24].

4. Surface Analyses

SEM images of the untreated and plasma-treated samples were taken to clarify the surface modification of the dyed polyester fibers due to the plasma treatment.

FESEM: JEOL JSM-7600F, produced by JEOL Ltd.-Tokyo-Japan at the Laboratory of Electron Microscopy and Microanalysis (BKEMMA) (Advanced Institute for Science and Technology (AIST), HUST, Hanoi, Vietnam) was used for these tests. The condition of SEM was at U = 5 kV, X (magnification) = 3500 and 50,000. All the samples were coated with platinum prior to observation by SEM.

##### 2.2.2.2. Characterization of the Control and Flame-Retardant-Treated Dyed Polyester Fabric

5. Flammability test

The vertical flammability test method ASTM D 6413:2015 was used for evaluating the flammability of the untreated and flame-retardant-treated samples after washing (FRT-W5 samples). The Vertical Flammability Chamber HQ-980-DMF VINA (Hanoi, Vietnam) was used.

The LOI value of the untreated, FRT samples and FRT samples after washing were also measured in accordance with the ASTM D 2863-00 standard method using Qinsun Limiting Oxygen Index Tester—F101D (Shanghai, China).

6. Shrinkage of Fabric due to High Temperature Curing

Since polyester is a thermoplastic fabric, the heat received from the plasma treatment and from the FR treatment can affect the size of the fabric. Therefore, the shrinkage of the fabric after plasma treatment and after FR treatment has been determined as follows:

Sample preparation for measuring the shrinkage of fabrics due to plasma treatment: To prepare for plasma processing, fabric samples were cut to a width of 35 cm and the required length. The sample length was selected according to the warp direction of the fabric. Squares measuring 30 cm × 30 cm were marked along the length of the samples (the sides of the square were 2.5 cm from the edges of the samples). The plasma-treated samples were conditioned for 24 h, then the dimensions of the squares were remeasured to determine the fabric shrinkage according to Equation (3).

For flame-retardant treatment, fabric samples were prepared with dimensions 35.0 cm × 35.0 cm. On each fabric sample, a square was marked with the size of 30.0 cm × 30.0 cm (the sides of the square were 2.5 cm from the edges of the sample). All fabric samples had been marked in the warp and weft directions. The FRT samples were conditioned for 24 h, and then the dimensions of the squares were remeasured to determine the fabric shrinkage according to Equation (3).

The sizes of squares before and after the treatments were also measured by a ruler with an accuracy of millimeters.

Shrinkage due to the treatment is calculated according to Equation (3).

(3) $$\mathrm{Shrinkage}\;\left(\%\right)=\frac{30-D\;\left(\mathrm{cm}\right)}{30}\times 100$$

7. Surface Analyses

Scanning electron microscope (SEM) and energy-dispersive spectroscopy (EDS) were conducted for the surface morphology observation and elemental analysis of the dyed polyester fabrics before and after flame-retardant treatment with and without washing. All the samples were coated with platinum prior to observation by FESEM: JEOL JSM-7600F at the Laboratory of Electron Microscopy and Microanalysis (BKEMMA) (Advanced Institute for Science and Technology (AIST), HUST, Hanoi, Vietnam). The condition of SEM was at U = 5 kV, X (magnification) = 3500 and 50,000. The EDS test was also conducted by this machine.

## 3. Results

### 3.1. Effect of the DBD Plasma Treatment on Properties of the Dyed Polyester Fabric

#### 3.1.1. The Tensile Strength

The dyed PET fabric was exposed by a different plasma time of 15–90 s. The tensile strength of the plasma treated samples in comparison with the control fabric are presented in Table 2.

Table 2 shows that the highest value of the standard deviation is 39 N, which is equivalent to 2.8% of the tensile strength. This means all the obtained results are reliable because of all the small standard deviations. Thus, these results have the validity for further analysis.

Based on the shown results in Table 2, with plasma exposure time from 15 to 60 s, the tensile strength of plasma treated fabric in both warp and weft direction has little difference compared with control fabric. It can be considered that a plasma power of 1 W/cm^2^ for a period of 60 s has not been able to cause obvious effects on the tensile strength of this fabric. However, when the plasma contact time was extended to 90 s, the tendency of its influence on the tensile strength of the fabric in both warp and weft directions was more obvious. Fabric tensile strength is reduced by up to 4.24% in the warp direction and 2.95% in the weft direction. This trend was also observed in publication [25], the tensile strength of polyester fabrics made from filaments was slightly reduced by plasma treatment, while under the same effect, polyester fabrics made from spun yarns increased slightly. The reason for the increase in tensile strength of polyester fabrics made from spun yarn is explained in publications [23,25]. As for polyester fabrics made from filaments, a long DBD plasma treatment generates deep cracks on the PET fiber surfaces leading to reduced tensile strength of the fibers [25].

In our study, this phenomenon will also be explained through with the SEM images of the samples in the next Section 3.1.4.

#### 3.1.2. The Capillary Property of Fabric

The dyed PET fabrics which were treated by plasma with different times: 0, 15, 30, 60 and 90 s, also measured their capillaries on both warp and weft directions. The results of the wicking height of these samples are shown in Table 3 and Table 4.

Table 3 and Table 4 show that all the results are with small standard deviations such that they are sufficiently reliable for further analyses. The wicking heights in both warp and weft directions of all plasma-treated samples appear to have increased compared with the untreated sample. The longer the plasma exposure time is, the higher the increase in wick height is. However, it seems that this increase was rapid with a plasma exposure time of 15 s to 30 s; it slowed down when plasma exposure time was longer, from 30 s to 90 s.

This phenomenon has also been observed for polyester fabric after low pressure plasma treatment using a mixture of nitrogen, oxygen and argon [26] and after DBD treatment [23,25]. These phenomena have been explained by the appearance of new polar functional groups containing oxygen under the influence of the plasma environment, as confirmed by XPS analysis of the plasma treated PET samples [23,25,26,27]. Work [23] also suggested that the surface roughness and especially the appearance of grooves on the fiber surface due to plasma action could partly explain the increase in the wicking height of the PET fabric. In our research, this circumstance will also be discussed with the SEM images of the samples in the next section.

#### 3.1.3. Color Measurement Results

The color parameters of the samples and the color differences between the control and plasma-treated samples given by Ci4200 Spectro-Photometer are shown in Table 5.

Table 5 shows the color parameters of the control sample corresponding to the light gray color. Table 5 also shows that DE between control and plasma-treated samples increases with plasma exposure time. The smallest DE_CIELab_ value is 1.68 and the biggest is 3.42, corresponding to plasma exposure time of 15 and 90 s. That indicates that the plasma treatment had an effect on the shade of the dyed PET fabric. The DE_CIELab_ values between the control and P15, P30 and P60 samples are less than 2, this value between P90 and control sample is 3.45 (greater than 2 and less than 3.5). Thus, according to the assessment specified in document [22], the color difference between P15, P30 and P60 and the reference sample is small, obvious only to the trained eye, while the difference between sample P90 and control sample is medium, also obvious totrained eye.

**Figure 6:**
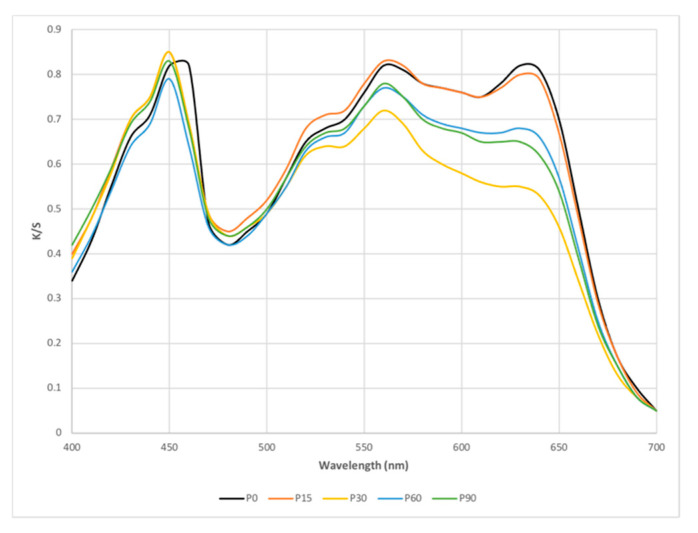
K/S value of untreated and treated samples. The black curve is for the untreated dyed polyester fabric (P0), and orange, yellow, light blue and green are for the respectively plasma-treated dyed polyester fabrics of 15 s (P15), 30 s (P30), 60 s (P60) and 90 s (P90).

The color strength (K/S) of samples (following the procedure described in Section 2.2.2.1) given by Ci4200 Spectro Photometer, over the range of 400 to 700 nm are presented in Figure 6.

Figure 6 shows the absorption peak of the plasma-treated samples observed at 450 nm while this peak of the control sample is around 460 nm. Thus, there is a slight color change in the plasma treated samples.

The color intensities of the samples were assessed by their maximum color strength value (K/S_max_). Figure 6 also shows that the K/S_max_ values of the control and P15 samples are almost the same. However, these values of the P30 and P60 samples have been slightly reduced. Sample P90 has the smallest K/S_max_ value. It can be said that the dyed PET fabric has been slightly faded due to plasma treatment. This phenomenon was also observed in study [28], when the dyed knitted cotton fabric was treated with a plasma jet. Thus, it is also necessary to consider the color factor of dyed fabrics when choosing plasma treatment conditions for fabrics.

#### 3.1.4. Surface Analysis—Scanning Electron Microscope (SEM)

**Figure 7:**
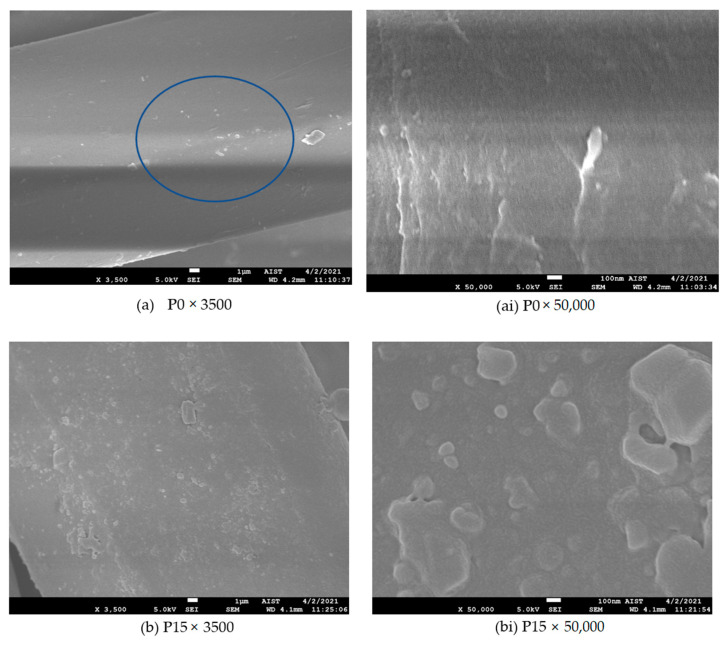

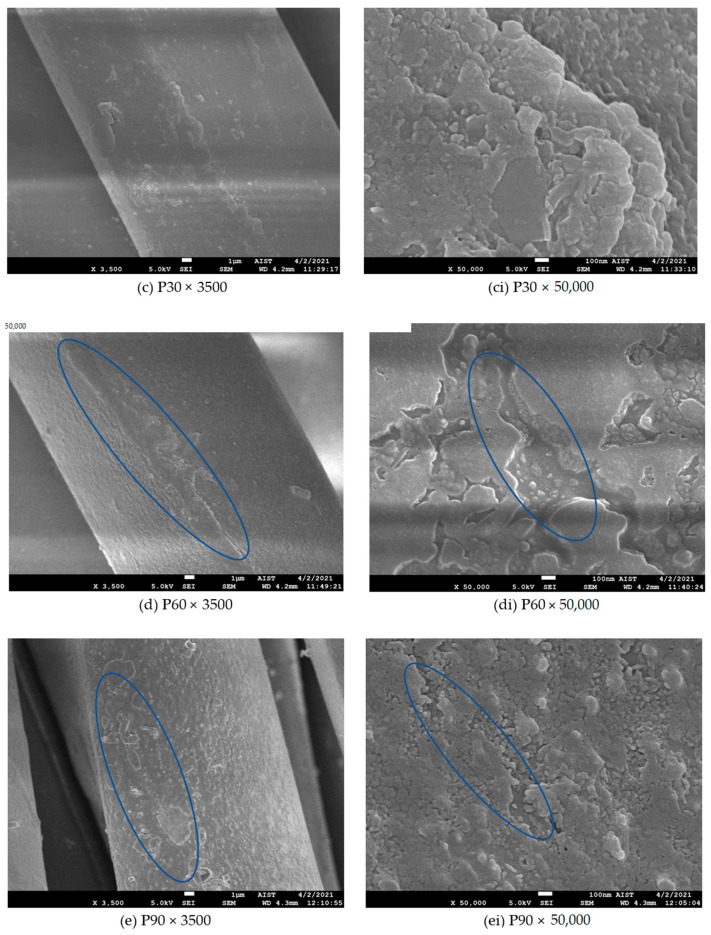
SEM images of the dyed PET fibers: (**a**,**ai**), images of the untreated fiber (P0); (**b**–**e**) and (**bi**–**ei**), images of plasma treated dyed PET fibers for 15 (P15), 30 (P30), 60 (P60) and 90 (P90) seconds with 3500× and 50,000× magnification, respectively.

SEM images of the untreated and plasma-treated dyed polyester fibers for 15, 30, 60 and 90 s (×3500 times) are illustrated in Figure 7a–e, respectively. Figure 7(ai–ei) shows the surface morphology of the controlled polyester fiber and the plasma-treated dyed fiber for 15, 30, 60 and 90 s (at high magnifications, ×50,000 times).

Figure 7a shows the fairly smooth surface apart from foreign matter that may be dispersed dye particles. It is not much because the fabric color is light gray; thus, the dye concentration is low. At 50,000× magnification (Figure 7(ai)), the fiber surface looks slightly rough. This may be the result of surface modification due to dyeing at 130 °C for 40 min. The complete opposite, all the plasma-treated dyed polyester fibers became harsh (Figure 7b–e). Besides, the surface morphology of the plasma-treated dyed polyester fibers also differs corresponding to plasma exposure time. With the 3500× magnification, we can easily see that the level of the roughness on the surface of the treated fibers is increased in direct proportion to plasma exposure time. When observing these surfaces at 50,000× magnification, the surface morphologies are apparently observed: while nodules (Figure 7(bi)) of different sizes appear unevenly on the 15 s plasma-treated dyed fiber surface, the scabrous layers (Figure 7(ci)) contribute densely to the 30 s plasma- treated dyed fiber surface; furthermore, with 60 s plasma treatment, fairly deep superficial lesions appear, in particular, they tend to form grooves on the fiber surface (Figure 7(di)). This phenomenon is consolidated for 90 s plasma treatment (Figure 7(ei)). In our previous work [23], a similar phenomenon was also observed for undyed polyester fabric made from staple fibers. It seems that the dye particles already present in the fiber structure did not affect the effect of plasma on PET fiber surface morphology. Thus, from the above SEM images, it can be assumed that there are surface lesions for the plasma treated fibers, which may be the cause of the decrease in tensile strength of the fabric when plasma exposure time is too long (90 s). Conversely, the observed surface roughness, in particular, the appearance of grooves on the fiber surface also contributed to an increase in the wicking height of the fiber. Furthermore, Kamel et al. [11] suggested that the rough surface of polyester fibers due to DBD treatment led to more physical loosening of the microstructure of the fabric. This can also contribute to the increased capillary of the fabric.

### 3.2. Effect of the Plasma Treatment on the Properties of the FRT Dyed PET Fabric

**Figure 8:**
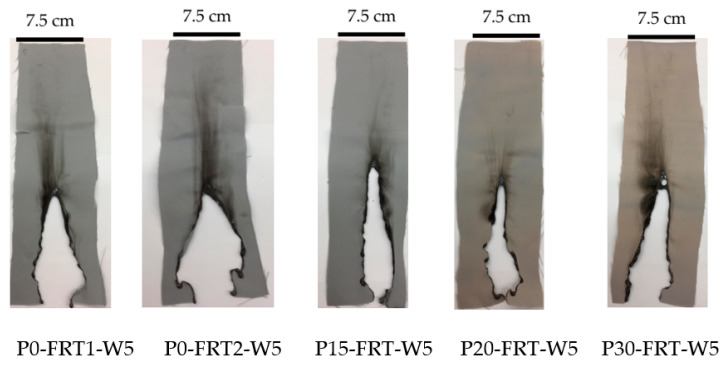
Photographs of samples after vertical flammability test according to ASTM D 6413.

From the results of Section 3.1, the following observations can be made: Based on the tensile strength and color parameters of the plasma treated fabrics, the plasma treatment time should not exceed 60 s. According to their wicking height, plasma contact time can be extended up to 90 s; however, the most effective plasma contact time for wicking height is only 30 s because the rate of increase in wicking height slows down after 30 s. From these results, 3 plasma treatment options for fabrics before FR treatment were selected at 15, 20 and 30 s. Afterward, the plasma-treated samples were further flame-retardant treated with the same process to find the most effective plasma treatment time. The properties of these three FRT fabrics were compared with two FRT fabrics without plasma pretreatment. The results of testing the flame-retardant characteristics of FRT samples and their shrinkage due to the plasma and curing steps are presented in Table 6. The images of FRT samples after 5 washing cycles and after vertical flammability test according to ASTMD 6413, are presented in Figure 8.

**Figure 9:**
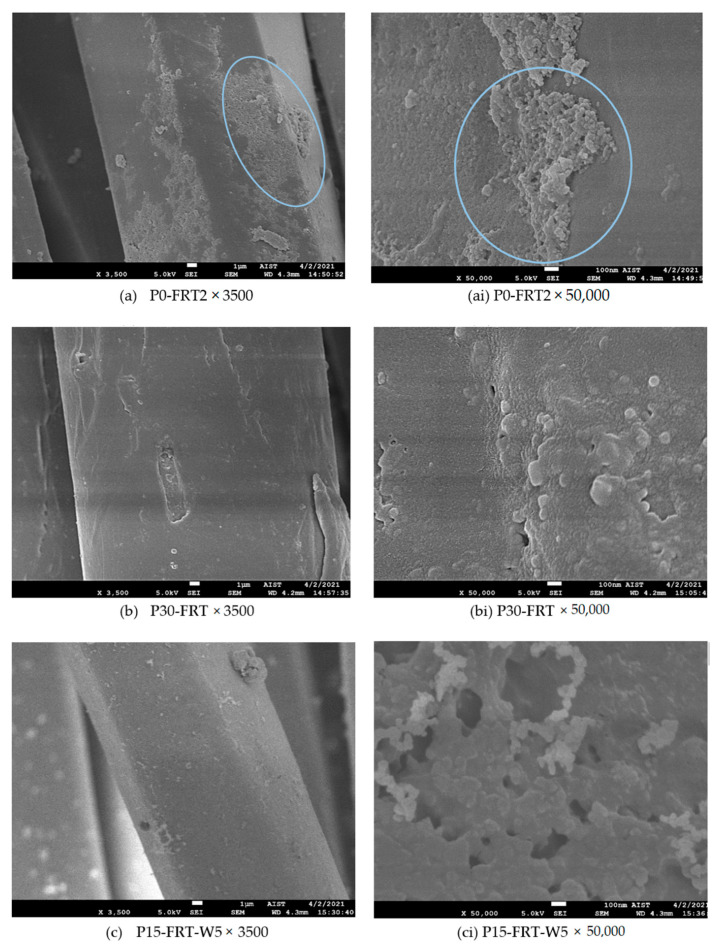

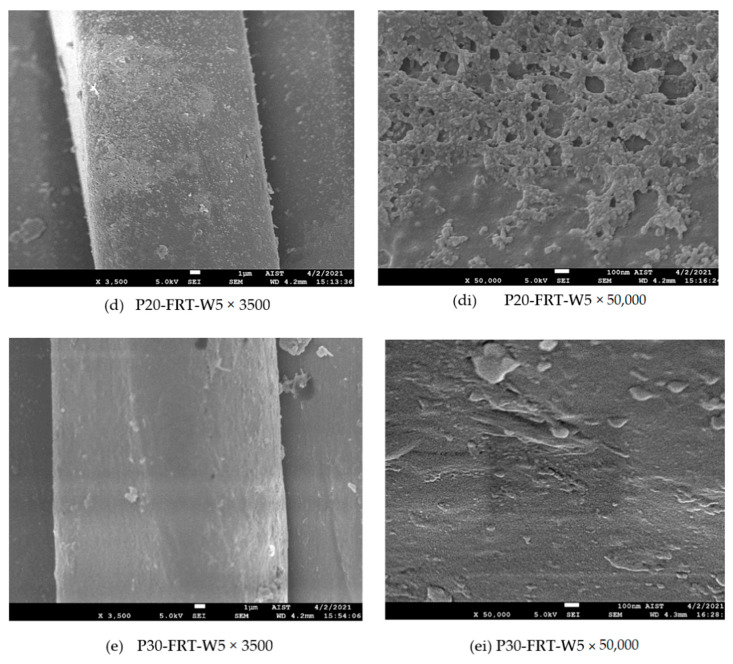
SEM images of PET fibers after flame-retardant treatment (**a**,**b**) and after being washed five times (**c**–**e**) at 3500×; (**ai**–**ei**) SEM images with 50,000× magnification of the samples corresponding to Figure 10a–e.

*Flammability of the FRT samples*: The results of the vertical flammability test of the fabric according to ASTM D 6413 standard show a clear difference between the control and the FRT samples after 5 washing cycles. Table 6 shows that in the vertical flammability test, after the flame was removed, the control sample continued to burn for up to 18.1 s while the AF of the FRT samples after 5 wash cycles was only between 0 and 1.5 s. It can be said that all the FRT samples are good durable flame-retardants. Thus, based on the AF value of the FRT samples after 5 washing cycles, it is not possible to clarify the effect of the curing time or plasma treatment time on their flame retardancy and their flame-retardant durability. However, in observing Figure 9, it can be seen that there is a difference in the burning behavior of the two sample groups. It seems that the plasma pre-activated FRT samples only burned vertically. Meanwhile, the FRT samples without plasma pretreatment burned in both directions. Besides, the LOI values of the FRT samples after treatment and after washing also show, more clearly, the effect of plasma pretreatment on the FR treatment for dyed polyester fabric. Table 6 shows that the LOI value of the dyed PET fabric (P0) is only 20.5%, this result is completely consistent with the flammability of polyester fabric in general [29]. All the FRT samples have high LOI values. The highest LOI value of 34.1% corresponds to the sample cured at 190 °C for 240 s without plasma pretreatment (P0-FRT1). Flame-retardant fabric samples that were cured at 190 °C for 120 s without plasma pretreatment (P0-FRT2) or with plasma pretreatment for 15, 20 and 30 s (P15-FRT, P20-FRT, P30-FRT) do not have considerably different LOI values (33.3, 33.3, 33.5 and 33.5%). Therefore, extending the curing time from 120 to 240 s increased the LOI value of the fabric from 33.3% to 34.1%, while plasma pretreatment of 15 to 30 s also increased the LOI value of the fabric from 33.3% to 33.5%. Moreover, the LOI values of FRT fabric samples after 5 washing cycles show that all 3 samples pretreated with plasma before flame-retardant treatment have LOI values from 30.0% to 30.8% compared to 28.0% and 28.6% of the samples without plasma pretreatment.

Thus, the flame retardancy of all FRT fabrics was reduced after being washed five times. However, this phenomenon occurred more easily for samples without plasma pretreatment. It seems that plasma pretreatments have really allowed to improve the wash durability of the FRT fabric’s fire resistance. This tendency has also been observed in work [4].

*Shrinkage of the FRT samples:* The results in Table 6 show that the dimensions of the PET fabric were not changed due to heat during plasma treatment. However, after the curing step, the fabric dimensions in both warp and weft directions of all samples were reduced, which happened more in the weft direction. Although all samples were cured at 190 °C, sample P0-FRT1 had the highest shrinkage (1.43% in the warp direction and 7.14% in the weft direction). Sample P30-FRT had the lowest shrinkage (0% in the warp direction and 4.29% in the weft direction). Thus, the effect of plasma pretreatment on reducing fabric shrinkage during curing was only apparent when the plasma exposure time was up to 30 s. It can be said that these results are also consistent with the LOI values of the FRT fabric samples after 5 washes. This phenomenon may be related to the surface modifications of polyester fiber by DBD treatment, which is discussed in Section 3.1 (wick height (Table 3 and Table 4) and the SEM images of plasma-treated polyester fabrics (Figure 7(b,bi,c,ci)). This relationship can be explained as follows.

About the effect of DBD plasma treatment on the microstructure of PET fabric, Kamel et al. [11] and Shahidi et al. [30] suggested that the etching effect is caused by the bombardment of ions/electrons in plasma on the surface of PET fabric, causing surface roughness. Thus, this rough surface led to more physical loosening of the microstructure of the fabric. Furthermore, X-ray diffraction measurements [11,31] also showed that the crystallinity of polyester fabrics was reduced by DBD plasma treatment. Dave et al. [9] used the FTIR spectrum to show the amorphization of polyester fabrics by DBD plasma treatment. It appears that amorphization occurred when polyester fibers were treated with DBD plasma, resulting in a looser, more porous microstructure. Regarding the change of chemical structure, works [23,25,26] also showed the appearance of new polar functional groups containing oxygen for polyester fibers under the influence of a plasma environment.

It is assumed that these chemical and physical changes both occurred in the DBD plasma-treated polyester fibers in this study (Table 3 and Table 4 and Figure 7). How did they affected the flame retardant treatment? In three stages of pad-dry-cure, the flame retardants were adsorbed on the surface of the PET fibers by the padding. Next, a small amount of flame retardant may have been absorbed by the PET fibers at 120 °C for 180 s of drying. This amount of absorption depends on the microstructural properties of the polyester fiber. The looser and more porous the microstructure, the higher the amorphous ratio, and the more flame retardant absorbed by the fibers. Finally, flame retardants on PET fibers were cured at 190 °C for 120 or 240 s. Non-curing flame retardants might have been removed during rinsing. The LOI value of FRT samples is related to the amount of flame retardant present in the sample (amount that has been cured, including both the amount adsorbed and the amount absorbed). However, during washing cycles, flame retardants that are only adsorbed on the surface of polyester fibers (including when cured) can be washed away gradually over washing cycles. Flame retardant, if already absorbed into the polyester fiber structure, is unlikely to be washed away in the washing cycle at 40 °C.

Thus, sample P0-FRT1 has the highest LOI value (Table 6) as it was cured at 190 °C for 240 s and thus has the highest amount of cured flame retardant. However, samples P15-FRT, P20-FRT and P30-FRT may have a higher amount of flame retardant absorbed due to their looser, more porous microstructure and higher capillary capacity (Table 3 and Table 4 and Figure 7). Therefore, after being washed five times, these samples have higher LOI values than samples P0-FRT2, including samples P0-FRT1 (Table 6). It is possible that the flame retardant molecules absorbed by the polyester fiber microstructure occupied part of the free volume of the amorphous region of the fiber. This limited the heat shrinking of the fabric during curing. The lower heat shrinkage of the P30-FRT sample compared with other samples cured under the same conditions can be explained as follows: The results in Table 3 and Table 4 and Figure 7 suggest that the P30 sample may have a more porous microstructure than the plasma-untreated sample (P0), and it may also be more porous than that of the plasma-pretreated samples but with a shorter plasma exposure time (P15 and P20). Thus, due to its more porous microstructure, more flame retardants can be absorbed by it. This may help it to limit the shrinkage during curing at 190 °C.

These phenomena will be further elucidated by surface analyses of FRT fibers in the next section.

### 3.3. Results of the Surface Analysis of Flame-Retardant Treated Fibers

The SEM images and EDS spectra of the samples after FR treatment and after being washed for five cycles were used to clarify the results on the FR properties of the fabric (Section 3.2).

SEM images of the P0-FRT2 (cured at 190 °C for 120 s without plasma pretreatment) and P30-FRT (cured at the same condition with plasma pretreatment for 30 s) (×3500 times) are illustrated in Figure 9a,b. Figure 9(ai,bi) shows the surface morphology (at high magnifications, ×50,000 times).

**Figure 10:**
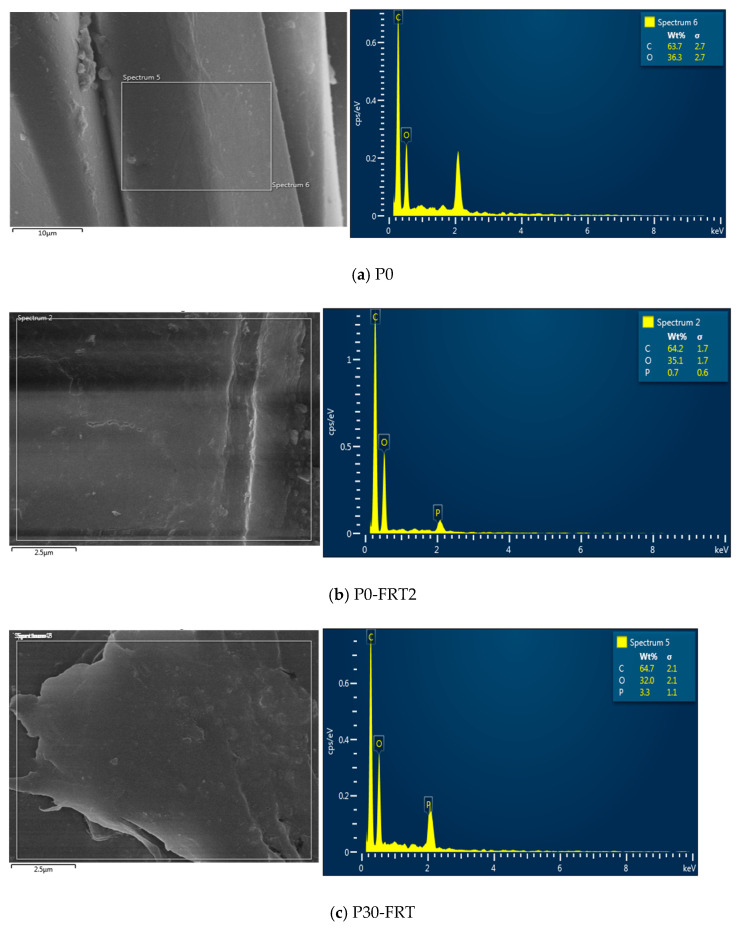

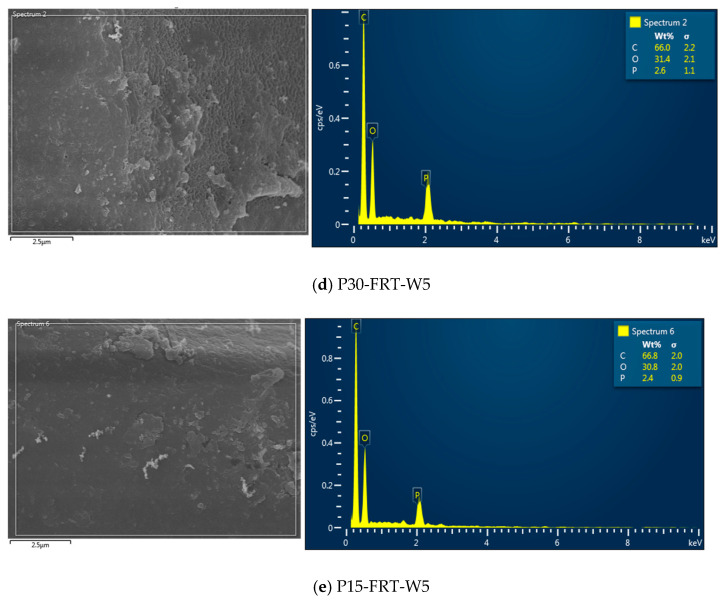
EDS spectra of the samples: (**a**) control (P0); (**b**) P0-FRT2; (**c**) P30-FRT; (**d**) P30-FRT-W5; and (**e**) P15-FRT-W5.

Observing Figure 9a,b, it shows that there are coatings on the fiber surface. Comparing these Figures with Figure 7a–c (control and plasma treated samples), it can be seen that these coatings appear only after the sample was being treated with flame retardants. Examination of the EDS spectra of these samples (Figure 10b,c) shows that phosphorus pick is present while the EDS spectra of the control sample (Figure 10a) shows only carbon and oxygen picks. Thus, these coatings are flame retardants that have been retained on the fiber surface by FR treatment. However, Figure 9(bi) show that the flame retardants completely cover the fiber surface (the roughness caused by plasma treatment is no longer visible (Figure 7c)). While Figure 9(a,ai) shows that the flame retardants cover only part of the fiber surface. Thus, with the same FR treatment condition (curing at 190 °C for 120 s), the plasma treated fibers could retain more flame-retardant than the non-plasma treated fibers. Furthermore, the SEM images of these two samples are also consistent with their LOI values (Table 6). Figure 9(c,ci,d,di,e,ei) shows that after five washing cycles, the coatings still cover the flame-retardant fibers with plasma pretreatment. Comparing Figure 9(ei) with Figure 7(ci), it shows that the rough surface due to plasma treatment (Figure 7(ci)) was completely covered with flame retardant.

To further clarify the effects of plasma pretreatment on the FR treatment for the dyed PET fabrics, the EDS spectra of several samples will be analyzed in the next section.

The control sample (P0), the FRT samples (P0-FRT2, P30-FRT) and the FRT samples after being washed for five cycles (P15-FRT-W5 and P30-FRT-W5) were measured, including the atomic content of various elements (C, O and P) to show the differences of phosphorus value, which contributes to the flame-retardance properties of the samples.

The EDS spectra of these samples are shown in Figure 10 inn which Figure 10b–e shows the EDS spectra of the samples P0-FRT2, P30-FRT, P30-FRT-W5 and P15-FRT-W5, respectively. Figure 10a shows the EDS spectrum of the control sample (dyed PET fabric).

In Figure 10a–e: On the left, it shows the area where the EDS was taken on the sample, and on the right, respectively. It is the EDS spectra which shows the atomic content of C, O and P. The atomic content of C, O and P in the samples that were determined from the EDS spectra are shown in Table 7.

As expected, the controlled sample (P0), which was not treated by FR solution, had a major percentage of carbon and oxygen atoms and no peaks P on its EDS spectrum (Figure 10a). On the EDS spectra of the FRT samples and themselves after being washed five times (Figure 10b–e), there is a phosphorus peak next to the carbon and oxygen peaks. This indicates the presence of flame retardant on these samples.

In Table 7, sample P0 (control sample), there are only carbon and oxygen. While sample P0-FRT2 (flame retardant fabric without plasma pretreatment) has the lowest phosphorus content of only 0.7%, sample P30-FRT (fire retardant fabric with plasma pretreatment for 30 s) has a phosphorus content of 3.3%. This result again shows that the plasma treated samples are able to retain more flame retardant than the non-plasma-treated samples when they were treated with flame-retardant under the same conditions. The phosphorus content of the flame-retardant fabric samples with plasma pretreatment for 30 and 15 s after being washed five times is 2.6% and 2.4%, respectively. Comparison of phosphorus content of the sample before washing (3.3%) and its own after washing (2.6%), shows that flame-retardants were removed from the flame-retardant sample during washing cycles. This result is also consistent with the LOI value of the P30-FRT sample before and after five wash cycles (Table 6). These may be flame retardants that have been adsorbed on the fiber surface. Flame retardants absorbed by polyester fibers can be difficult to remove during washing at 40 °C. Figure 9(e,ei) show that after five washing cycles, the fiber surface is still mostly covered with flame retardants.

## 4. Discussion

We assume that the behaviors of dyed polyester fibers in plasma are similar to those of undyed polyester fibers [4,23,24,30]. In the DBD plasma environment, when excited and energetic plasma species (ions, radicals, electrons and metastable) are bombarded onto the polyester surface, they can induce physical and chemical modifications for the polymer surface. The bombardment of ions/electrons in the DBD plasma can create the reactive species on the surface of treated sample. These reactive species may react with oxygen from the air, resulting in more oxygen-containing polar groups on the polyester surface after plasma treatment [4,23,27]. Furthermore, chemical etching occurs in chemically reactive types of plasma. During etching reaction, weight loss of the substrate occurs, and the topmost layer of the substrate is stripped off [16]. Moreover, the microstructure of polyester fiber has also been changed, and under the effect of DBD plasma, the fiber crystallinity has been reduced [9,11,31]. As a result, the fibrous structure can become looser and more porous. The increase in fiber surface roughness with plasma treatment time (Figure 7) is a clear demonstration of this surface modification. It was these physical and chemical modifications that led to the continued increase of the wicking hydrophilicity of fabric with plasma treatment time (Table 3 and Table 4). However, plasma etching and weight loss, if taken too long, can also lead to damage to the fabric, such as color change or decrease in tensile strength (Table 2 and Table 5). Under the conditions of this study, the plasma treatment time should not exceed 60 s such that the color of the fabric is not considerably changed, and the tensile strength of the fabric is not reduced.

In this study, it is the above-mentioned physical and chemical changes that make the FR treatment of plasma-treated polyester fabrics more efficient. The looser, more porous morphological structure may be allowed for the plasma-treated polyester fibers to capture more flame retardants, and the flame retardants can more easily penetrate the fiber structure.

## 5. Conclusions

From the research results, the following conclusions can be drawn:

(1) DBD plasma pretreatment for dyed PET fabrics can be a suitable surface modification method to make subsequent functional finishing more efficient;

(2) In this study, DBD plasma pretreatment at 1 W/cm^2^ for 15 s allowed the fabric wick height to increase to 12% in the warp direction and 24% in the weft direction. If further extended the treatment time to 90 s, the capillary of the fabric would increase to 28% and 61%, respectively. However, plasma treatment time of longer than 60 s also causes obvious color change and decrease in tensile strength of this dyed polyester fabric;

(3) With plasma pretreatment at 1 W/cm^2^ in 30 s, the subsequent flame-retardant treatment can be cured at 190 °C for 120 s and has a better flame-retardant resistance after five washing cycles versus those of the fabric which was cured at 190° C for 240 s but without plasma pretreatment. In addition, plasma pretreatment has also helped to reduce the heat shrinkage of polyester fabrics during curing;

(4) The surface modification of fabrics due to plasma treatment is clearly shown by SEM images of plasma treated PET fibers with magnifications of 3500 and 50,000 times. SEM images of fabrics treated with flame-retardant, their own after five washing cycles, and their EDS spectra are consistent with their LOI values;

(5) The efficient flame-retardant treatment for dyed polyester fabrics with a plasma pretreatment time of only 30 s and curing time of only 120 s is a novelty of this research. This is also a favorable condition for the process to be industrially deployed;

(6) However, the limitation of this study is that it has not mentioned the effect of plasma pretreatment on the required amount of flame-retardant. This content will be covered by us in future studies.

## Figures and Tables

**Table 1:** The experiments with variations of the plasma exposure times and flame-retardant finishing.

Exp. Name	Curing Temp (°C)	Curing Time (s)	Plasma Exposure Time (s)
P0-FRT 1	190	240	0
P0-FRT 2	190	120	0
P15-FRT	190	120	15
P20-FRT	190	120	20
P30-FRT	190	120	30

**Table 2:** The Tensile strength of the samples according to plasma exposing time.

Sample	in Warp Direction		in Weft Direction	
	Cal. F_MAX_ (N)	MSC (%)	Cal. F_MAX_ (N)	MSC (%)
P0	1932 ± 5	-	1456 ± 11	−
P15	1901 ± 9	−1.60	1457 ± 24	0.07
P30	1969 ± 10	1.92	1466 ± 21	0.69
P60	1967 ± 11	1.81	1444 ± 6	−0.82
P90	1850 ± 17	−4.24	1413 ± 39	−2.95

**Table 3:** The Wicking height of the samples in warp direction after 30 min according to plasma exposing time.

Sample	Wicking Height of Samples in Warp Direction (cm) Measured after					
	5 min	10 min	15 min	20 min	25 min	30 min
P0	6.8 ± 0.3	9.3 ± 0.3	10.7 ± 0.4	11.7 ± 0.4	12.3 ± 0.3	12.6 ± 0.4
P15	8.2 ± 0.2	10.5 ± 0.3	11.7 ± 0.1	12.7 ± 0.3	13.4 ± 0.3	14.1 ± 0.3
P30	8.9 ± 0.3	11.4 ± 0.3	12.6 ± 0.2	13.9 ± 0.1	15.5 ± 0.1	15.9 ± 0.1
P60	8.8 ± 0.2	11.2 ± 0.2	13.4 ± 0.3	14.1 ± 0.2	14.8 ± 0.2	15.8 ± 0.2
P90	9.8 ± 0.2	12.8 ± 0.3	14.2 ± 0.5	14.9 ± 0.0	15.5 ± 0.1	16.1 ± 0.1

**Table 4:** The Wicking height of the samples in weft direction after 30 min according to plasma exposing time.

Sample	Wicking Height of Samples in Weft Direction (cm) Measured after					
	5 min	10 min	15 min	20 min	25 min	30 min
P0	5.2 ± 0.3	6.5 ± 0.1	7.3 ± 0.1	8.1 ± 0.1	8.6 ± 0.2	8.9 ± 0.1
P15	5.9 ± 0.1	7.6 ± 0.2	8.9 ± 0.3	9.9 ± 0.1	10.5 ± 0.1	11.1 ± 0.1
P30	6.7 ± 0.3	8.4 ± 0.2	10 ± 0.4	10.9 ± 0.5	11.7 ± 0.4	12.3 ± 0.5
P60	6.5 ± 0.2	9.2 ± 0.2	10.5 ± 0.4	11.4 ± 0.2	12.1 ± 0.1	13 ± 0.0
P90	6.9 ± 0.2	9.7 ± 0.4	11.7 ± 0.6	12.7 ± 0.6	13.6 ± 0.7	14.3 ± 0.8

**Table 5:** Color parameters of the samples and DE between the control and plasma-treated samples.

Sample	L*	a*	b*	C*	h*	DE_CIELab_	DE_CMC_
P0	64.20	−2.88	−2.47	3.80	220.70	-	-
P15	64.55	−1.57	−1.49	2.16	-	1.68	1.89
P30	64.69	−1.76	−1.43	2.27	-	1.60	1.77
P60	64.63	−1.73	−1.28	2.15	-	1.71	1.91
P90	63.61	−0.75	0.13	0.76	-	3.42	3.95

L*, a*, b* are three components of color in the CIEL* a* b* space: L*—lightness, where 0 means black, and 100 is the maximum light intensity which is still visible without causing eye damage; a*—color in the green ÷ red field (−128, +127), b*—color in the blue ÷ yellow field (−128, +127). In the middle (a* = 0; b* = 0) only gray values exist. L*, C*, h* are three components of color in the CIEL*C*h* space (L*—lightness, C*—chroma(saturation), h*—hue). The CIEL*a*b* space is described in Cartesian coordinate, while the CIEL* C* h* space—in cylindrical coordinates. The relationships between their respective coordinates are therefore as follows: L* ≡ L*, C* = $\sqrt{{a*}^{2}+{b*}^{2}}$; h* = artan (b*/a*) [22].

**Table 6:** The flame-retardant characteristics of FRT samples and their shrinkage due to the treatment.

Sample	After Flame (AF) Time of FRT-W5 (s)	LOI of Sample (%)		Shringkage due to Plasma Treatment (%)		Shringkage due to Curing (%)	
		After Treatment FRT	After 5 Washing FRT-W5	Warp Direction	Weft Direction	Warp Direction	Weft Direction
P0	18.1	20.5	-	-	-	-	-
P0-FRT1	1.0	34.1	28.6	-	-	1.43	7.14
P0-FRT2	1.5	33.3	28.0	-	-	1.43	5.71
P15-FRT	0.0	33.3	30.0	0.0	0.0	1.43	5.71
P20-FRT	0.0	33.5	30.8	0.0	0.0	1.43	5.71
P30-FRT	1.0	33.5	30.3	0.0	0.0	0.00	4.29

**Table 7:** The atomic percentage of different elements (C, O and P) present in the samples were determined from the EDS spectra.

Sample	C (%)	O (%)	P (%)
P0	63.7 ± 2.7	36.3 ± 2.7	-
P0-FRT2	62.3 ± 1.7	35.1 ± 1.7	0.7 ± 0.6
P30-FRT	64.7 ± 2.1	32.0 ± 2.1	3.3 ± 1.1
P30-FRT-W5	66.0 ± 2.2	31.4 ± 2.1	2.6 ± 1.1
P15-FRT-W5	66.8 ± 2.0	30.8 ± 2.0	2.4 ± 0.9

## Data Availability

Not applicable.

## References

[1] Hatch K.L. (1993). Textile Science.

[2] Kundu C.K., Li Z., Song L., Hu Y. (2020). An overview of fire retardant treatments for synthetic textiles: From traditional approaches to recent applications. Eur. Polym. J..

[3] Üreyen M.E., Kaynak E. (2019). Effect of zinc borate on flammability of pet woven fabrics. Adv. Polym. Tech..

[4] Ömeroğulları Z., Kut D. (2012). Application of low-frequency oxygen plasma treatment to polyester fabric to reduce the amount of flame retardant agent. Text. Res. J..

[5] Bendak A., El-Marsafi S.M. (1991). Effects of chemical modifications on polyester fibres. J. Sci. Islam. Repub. Iran..

[6] Pitchai S., Moses J., Natarajan S. (2014). Study on the improvement of hydrophilic character on polyvinylalcohol treated polyester fabric. Pol. J. Chem. Technol..

[7] Natarajan S., Moses J.J. (2012). Surface modification of polyester fabric using polyvinyl alcohol in alkaline medium. Indian J. Fibre Text. Res..

[8] Dumecha B., Nalankilli G. (2017). Anionic dyeability of polyester fabric by chemical surfave modification. Int. J. Mod. Trends Sci. Technol..

[9] Dave H., Ledwani L., Chandwani N., Kikani P., Desai B., Nema S.K. (2013). Surface modification of polyester fabric by non-thermal plasma treatment and its effect on coloration using natural dye. J. Polym. Mater..

[10] Malinowska G. (2009). Effect of the corona discharge treatment of polyester fabrics on their adhesive properties. Fibres Text East Eur..

[11] Kamel M.M., El Zawahry M.M., Helmy H., Eid M.A. (2011). Improvements in the dyeability of polyester fabrics by atmospheric pressure oxygen plasma treatment. J. Text. Inst..

[12] Gabr B.G., Salem A.A., EL-Kholy G.A., Hassaballa A.E.S.S. (2016). Wettability and water vapor transfer rate of knitted garments utilizing non-thermal atmospheric pressure plasma. J. Am. Sci..

[13] Zhang C., Zhao M., Wang L., Qu L., Men Y. (2017). Surface modification of polyester fabrics by atmospheric-pressure air/He plasma for color strength and adhesion enhancement. Appl. Surf. Sci..

[14] Basuk M., Bait S., Maiti S., Adivarekar R.V. (2018). Management Properties and Drying Behavior of Polyester and Blend Fabrics for Sportswear Application. Curr. Trends. Fashion Technol. Text. Eng..

[15] Sun D., Kalia S. (2016). Surface modification of natural fibers using plasma treatment. Biodegradable Green Composites.

[16] Nguyen T.H., Vu T.H.K., Ngo H.T., Phan D.N. (2020). Application of Plasma Activation in Flame-Retardant Treatment for Cotton Fabric. Polymers.

[17] Zille A., Oliveira F.R., Souto A.P. (2015). Plasma treatment in textile industry. Plasma Process. Polym..

[18] Sohbatzadeh F., Farhadi M., Shakerinasab E. (2019). A new DBD apparatus for super-hydrophobic coating deposition on cotton fabric. Surf. Coat. Technol..

[19] Raslan W.M., Rashed U.S., El-Sayad H., El-Halwagy A.A. (2011). Ultraviolet protection, flame retardancy and antibacterial properties of treated polyester fabric using plasma-nano technology. Mater. Sci. Eng. C.

[20] Carosio F., Alongi J., Frache A. (2011). Influence of surface activation by plasma and nanoparticle adsorption on the morphology, thermal stability and combustion behavior of PET fabrics. Eur. Polym. J..

[21] Furlan T., Nešković I., Špička N., Golja B., Kert M., Tomšič B. (2019). Multifunctional Hydrophobic, Oleophobic and Flame-retardant Polyester Fabric. Tekstilec.

[22] Mokrzycki W.S., Tatol M. (2011). Colour difference ∆E-A survey. Mach. Graph. Vis..

[23] Kim T.N.T., Vu T.H.K., Vu T.N., Vu Manh H. (2021). The Effect of DBD Plasma Activation Time on the Dyeability of Woven Polyester Fabric with Disperse Dye. Polymers..

[24] Dave H., Ledwani L., Chandwani N., Desai B.D., Nema S.K. (2014). Surface activation of polyester fabric using ammonia dielectric barrier discharge and improvement in colour depth. Indian J. Fibre Text. Res..

[25] Gotoh K., Yasukawa A. (2011). Atmospheric pressure plasma modification of polyester fabric for improvement of textile-specific properties. Text. Res. J..

[26] Da Silva R.C.L., Alves C., Nascimento J.H., Neves J.R.O., Teixeira V. (2012). Surface modification of polyester fabric by non-thermal plasma treatment. J. Phys. Conf. Ser..

[27] Kerkeni A., Behary N., Perwuelz A., Gupta D. (2012). Dyeing of woven polyester fabric with curcumin: Effect of dye concentrations and surface pre-activation using air atmospheric plasma and ultraviolet excimer treatment. Color. Technol..

[28] Cheung H.F., Kan C.W., Yuen C.W.M., Yip J., Law M. (2013). Colour fading of textile fabric by plasma treatment. J. Text. Inst..

[29] Price D., Horrocks A.R., Kilinc F.S. (2013). Combustion processes of textile fibres. Handbook of Fire Resistant Textiles.

[30] Shahidi S., Ghoranneviss M., Wiener J. (2015). Improving synthetic and natural dyeability of polyester fabrics by dielectric barrier discharge. J. Plast. Film Sheet..

[31] Nithya S., Aranganayagam K.R. (2018). Characterization of atmospheric pressure plasma treated polyester fabrics. Rasayan J. Chem..

